# ShoRAH: estimating the genetic diversity of a mixed sample from next-generation sequencing data

**DOI:** 10.1186/1471-2105-12-119

**Published:** 2011-04-26

**Authors:** Osvaldo Zagordi, Arnab Bhattacharya, Nicholas Eriksson, Niko Beerenwinkel

**Affiliations:** 1 Department of Biosystems Science and Engineering, ETH Zurich, Mattenstrasse 26, 4058 Basel, Switzerland; 2 SIB Swiss Institute of Bioinformatics, Switzerland; 3 23andMe, Mountain View, CA 94043, USA

## Abstract

**Background:**

With next-generation sequencing technologies, experiments that were considered prohibitive only a few years ago are now possible. However, while these technologies have the ability to produce enormous volumes of data, the sequence reads are prone to error. This poses fundamental hurdles when genetic diversity is investigated.

**Results:**

We developed ShoRAH, a computational method for quantifying genetic diversity in a mixed sample and for identifying the individual clones in the population, while accounting for sequencing errors. The software was run on simulated data and on real data obtained in wet lab experiments to assess its reliability.

**Conclusions:**

ShoRAH is implemented in C++, Python, and Perl and has been tested under Linux and Mac OS X. Source code is available under the GNU General Public License at http://www.cbg.ethz.ch/software/shorah.

## Background

The advent of a new generation of high-throughput DNA sequencing technologies, known as deep sequencing or next-generation sequencing (NGS), has opened up new experimental approaches in basic, applied and clinical research. Among the applications that benefit from the enormous volume of data produced by NGS is the study of genetic diversity in heterogeneous samples. Genetic diversity has significant consequences, for example, in HIV infection. The set of diverse variants of the virus is responsible for disease progression and hampers efforts to develop effective therapies [1]. Other examples of biological systems where genetic diversity is equally important include bacterial communities [2] and cancer cells [3].

Traditional Sanger sequencing of a genetically diverse sample results in the consensus sequence of the population, in which low- frequency variants are not detected (the threshold being around 20%). This limitation is overcome in deep sequencing, which can be used to accurately quantify genetic diversity in a mixed sample, provided that sequencing errors are properly treated [4]. In many genetic analyses only a portion of the genome, e.g., a single gene, is isolated and PCR amplified. The sample is then sequenced on a NGS platform which performs hundreds of thousands of clonal sequencing reactions and produces a set of reads that is statistically representative of the diversity in the sample. The reads can be aligned to the reference sequence of the amplified region. In order to obtain a reliable estimate of the genetic variation, the confounding effects of sequencing errors must be taken into account [5].

**Figure 1:**
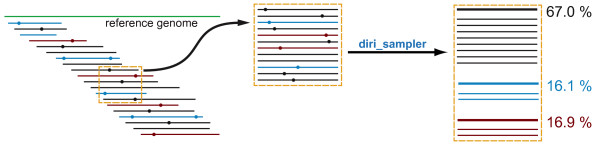
**Schematic view of the local haplotype reconstruction**. From the multiple sequence alignment of all reads, a window is defined and reads overlapping it are extracted and passed (in fasta format) to the program diri_sampler. The program returns (1) the inferred haplotype sequences, (2) their frequencies, (3) the set of corrected reads and (4) the full posterior probability of the reconstruction (not reported in the Figure). Same colour indicates reads originating from the same haplotype. Dots represent errors on the reads that are corrected in order to infer the haplotypes (thicker lines).

In order to correct sequencing errors and to estimate the population structure of a heterogeneous sample we developed ShoRAH (Short Read Assembly into Haplotypes). This software corrects sequencing errors by clustering all reads that overlap the same region of the genome of length approximately equal to the read length (window, see Figure 1). The consensus sequence of each cluster represents the original haplotype from which the erroneous reads were obtained. The number of reads associated with the cluster estimates the prevalence of the haplotype in the population. For many applications, this local diversity estimate is sufficient to draw important conclusions. For example, with current pyrosequencing reads of length about 500 bp, the protease gene of HIV, an important target of antiretroviral therapy, can be covered completely. Longer haplotypes sequences can be reconstructed from adjacent, overlapping reads, and the frequencies of these global haplotypes can then be estimated.

ShoRAH is a set of computational tools for inferring the local and global population structure from a set of reads obtained from a mixed sample in a single deep sequencing run.

We report here the main feature of the software, by giving an overview of its implementation, usage examples and a brief outline of the obtained results. We conclude by discussing the relevance of the software and possible future improvements.

## Implementation

**Figure 2:**
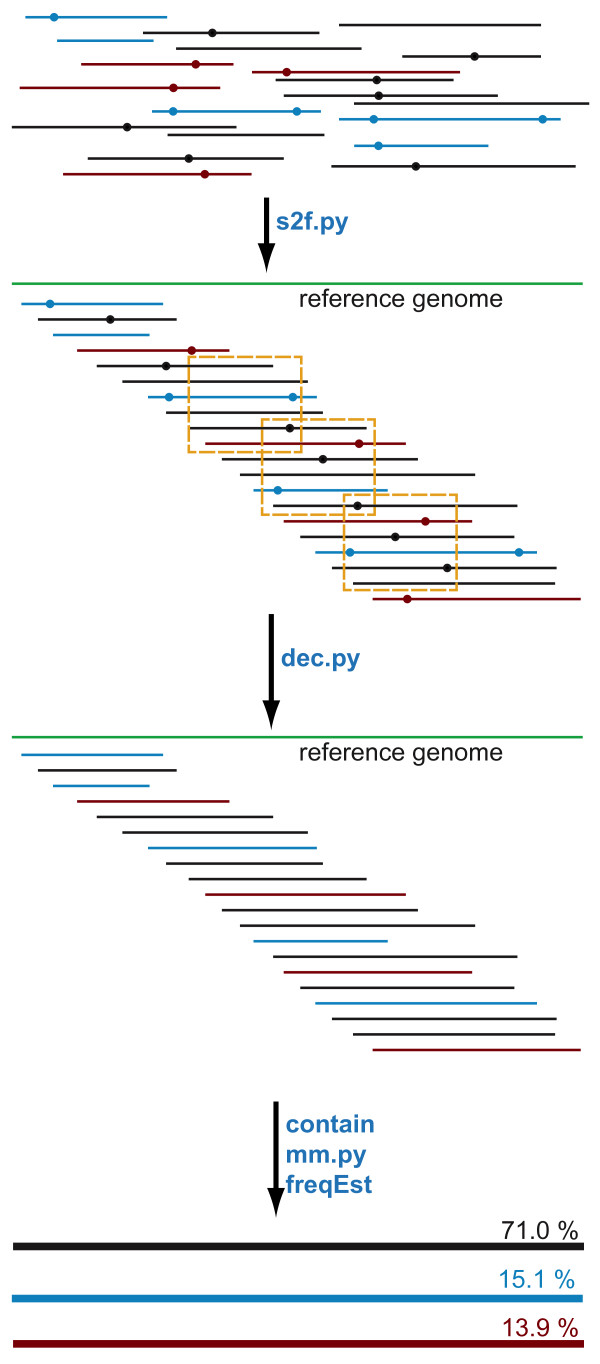
**Schematic view of the global haplotype reconstruction**. In the global analysis, users can start from the NGS reads and use the program s2f.py to produce a multiple alignment. Alternatively, one can use another tool to produce the alignment and enter the program workflow at a different point. The program dec.py constructs overlapping windows on this alignment and calls diri_sampler on each window for the local haplotype reconstruction. Then, it collects all results from the individual windows and corrects the reads. The set of corrected reads is passed to the programs contain, mm.py and freqEst to reconstruct the global haplotypes and estimate their frequencies.

The ShoRAH package is a collection of tools to estimate genetic variations from deep sequencing data by correcting sequencing errors, assembling reads and estimating their frequencies. It is implemented in C++, Python, and Perl and the source code is distributed under the GPL licence. Online documentation with installation and running instruction is available at https://wiki-bsse.ethz.ch/display/ShoRAH. Users can choose to run a local (Figure 1) or global analysis (Figure 2), depending on the question of interest and the available data. ShoRAH has been tested on reads generated by the 454/Roche FLX and the Illumina Genome Analyzer sequencing platforms.

Full analysis with ShoRAH includes four major steps that are performed by calling the wrapper shorah.py. These are: 1) alignment; 2) error correction (local haplotype reconstruction); 3) global haplotype reconstruction and 4) frequency estimation. Users can also choose to run only part of the analysis, for example stopping after the local reconstruction [4]. The input can be either a fasta file with NGS reads plus a reference sequence, or a multiple alignment of the reads in fasta format, if users wish to use their own alignment. The program s2f.py performs a pairwise alignment of all reads to the reference sequence and then builds a multiple sequence alignment (MSA) by padding every read with gaps found in the other pairwise alignments. More sophisticated approaches can be used in this step [6], but this method performs well in terms of quality and speed.

### Local analysis

The program dec.py considers a set of overlapping windows on the MSA and passes each to the program diri_sampler. Each run of diri_sampler estimates the diversity at the local level of the MSA window, the width of which should be approximately the average read length. This program employs a model-based probabilistic clustering algorithm to correct errors, infer haplotypes and their frequencies, and estimate, in a Bayesian fashion, the quality of the reconstruction by computing the full joint posterior probability distribution of all parameters of interest [7]. With this approach, neither independent estimates of the error rate nor of the number of haplotypes are needed in advance. Rather, they are estimated from the data. Two parameters can influence the outcome of this stochastic algorithm: the number of iterations and the hyper parameter *α*. Although there is no general rule to choose them, they have to be large enough in order to guarantee a proper mixing in the sampling. In particular, one has to avoid situations in which solutions with new clusters are never proposed (*α *too small). Nevertheless, the results hardly change over an interval of of several orders of magnitude. The online documentation contains further advice on how to tune the parameters.

In the default setting, dec.py constructs overlapping windows such that each position of the alignment is covered three times. The original reads are corrected following a majority vote based on these local reconstructions.

### Global analysis

The set of corrected reads is passed to the program contain in order to identify the set of unique reads of maximum length with respect to the substring relation. They are passed on to the program mm.py which implements the global reconstruction method. It employs a parsimony principle and computes a minimal set of haplotypes that explains all reads in the dataset [5].

Finally, the program freqEst implements an Expectation Maximization (EM) algorithm to estimate the frequencies of the reconstructed haplotypes by maximum likelihood [5]. The algorithm stops when the difference in the log-likelihood between two successive iterations is below a given threshold (EM converged) or when the number of iterations exceeds a maximum (EM not converged). Both parameters can be tuned (the default values are 10^-6 ^and 5000, respectively).

### Short reads

Platforms like Illumina Genome Analyzer produce datasets composed of more reads that are shorter than those produced with the 454 platform (up to 10^8 ^reads of 100 bp). For such very large datasets it is more practical to produce an alignment in SAM format (most NGS read mappers support this format), use the tool bam2msa.py to extract a MSA for a single window and simply run diri_sampler (local analysis) on this single file. Global haplotype reconstruction is increasingly difficult for long genomic regions and short reads, because of the difficulty and ambiguity of phasing variation at different sites. For a given read length, the solution of the global reconstruction becomes less stable for longer genomic regions, an effect that users should take into account in designing their experiments.

### Computational efficiency

The alignment step consists of pairwise alignments of all reads to the reference sequence. Its time complexity is proportional to the number of reads, the read length, and the length of the reference sequence.

The error correction step is run independently on each sequence window and, unlike the other components, is efficiently parallelized. The program diri_sampler implements a Gibbs sampler with time complexity per iteration proportional to the number of unique reads in the MSA window and to the number of clusters (i.e., haplotypes), which depends on the local diversity of the sample. The implementation of the Gibbs sampler is optimized by exploiting the fact that the number of unique reads is usually much smaller than the total number of reads. Moreover, the Hamming distances between sequences, which make up the sufficient statistics of the model, are computed in time independent of the sequence length and proportional to the distance [8]. This approach allows for analysing windows with up to 10^5 ^reads with a RAM usage on the order of gigabytes. The memory requirements depend mainly on the number of unique reads, or, in other words, on the expected diversity, the error rate and the width of the window. The global reconstruction program mm.py solves an instance of the maximum weight matching problem of time complexity at worst cubic in the total number of unique reads. Frequency estimation with the program freqEst is based on an EM algorithm. Its time complexity per iteration is the number of reads times the number of reconstructed haplotypes.

In practice, thanks to its parallel implementation, the error correction step was completed in less than 12 hours using 6 CPUs at 2.8 GHz for a large sample consisting of 40,000 pyrosequencing reads (average length 300 bp). freqEst completed the subsequent analysis in two days. The impact of mm.py and the wrapper shorah.py on total running time is negligible.

## Results and Discussion

### Local analysis

The local reconstruction performance of ShoRAH has been assessed using both simulated and experimental data. A heterogeneous sample was prepared by simulating reads from 10 different variants of the HIV-1 subtype B *pol *gene and mixing them at various proportions [7]. In general, the performance depends on the error rate of the sequencing process and on the frequencies and pairwise distances of the haplotypes in the sample. Haplotypes with frequencies as low as 0.1% were detected at a coverage of 5000 and their frequency correctly estimated [7].

A mixture with the same haplotypes used for the *in silico *experiment was prepared *in vitro *and the reads obtained with the Roche/454 Titanium platform were analysed. Similar results were obtained, inferring individual clones with frequencies as low as 0.1% with perfect sequence identity and good estimate of the frequency at a coverage of 6000. A five-fold decrease of the error rate, from 0.25% to 0.05%, was achieved [4].

A crucial advantage of clustering reads is the possibility to reliably detect co-occurrence of mutations when they are close enough to be captured by the same read. This information cannot be obtained with Sanger sequencing, and most software for NGS data only allows the detection of SNP at individual sites. ShoRAH was successfully used to reconstruct local haplotypes of the HIV-1 subtype B protease (*pol*) gene, an important target of anti-retroviral therapy, which can be entirely covered by a single 454 read [4].

### Global analysis

Global reconstruction and frequency estimation was evaluated on simulated error-free reads and real data as well [5]. Tests with error-free reads show that the performance of the global reconstruction depends on the number of present haplotypes, their diversity and the error rate of the machine, while being less affected by the number of reads.

### Short read data

Error correction and local haplotype inference was validated also on simulated and real Illumina data [8]. Haplotypes with frequency as low as 0.1% were detected and their frequency correctly estimated at a coverage of 5000.

Genetic diversity is also important in cancer and different variants in tumours samples can be detected by sequencing at very high coverage. Therefore, we used ShoRAH also in this setting. PCR amplicons were designed to cover regions of the human genome known to have a role in the development of rhenal cancer. Samples from tumour dissection were sequenced on these regions at high coverage on a Illumina GA (Gerstung *et al.*, in preparation). ShoRAH identified the co-occurrence of two SNPs at neighbouring sites of the *VHL *gene in a fraction of the sampled cells. The short distance between these two SNPs (53 bases apart) allowed 70 bp long Illumina reads to detect them simultaneously.

### Related work

Quantification of genetic diversity is one of the many possible applications of NGS technologies. For all applications, solid statistical models and efficient computational tools are prerequisites to fully exploit the produced data. Different models to assess genetic diversity have been presented in the last few years [9-12], but software implementing them is not yet available. There is a program for a related task, namely the assembly of reads from metagenomics studies [13]. While ShoRAH addresses the problem of identifying the presence of close haplotypes in a relatively short region of the genome by exploiting very deep coverage, in metagenomics studies one typically faces the problem of identifying very diverse genomes (from different species) that have been mixed together.

### Future work

Currently, the steps to reconstruct the population structure consist in 1) alignment, 2) error correction, 3) global haplotype reconstruction and 4) frequency estimation. Tools based on different models are used in these steps and this separation clearly has some limitations. For example, while the error correction is performed in a full probabilistic framework, only the most likely solution is used in the following step. Performing at least some of the steps in the same probabilistic model would likely be an advantage. Current efforts are aimed at developing a common framework for error correction and global haplotype reconstruction. Further, the software does not make full use of information from paired ends. Another possible improvement of the method might exploit the co-occurrence of variants on the same pair to reconstruct global haplotypes more reliably [12]. Currently, users can run ShoRAH on a set of reads obtained with paired end protocol, if they want to exploit the higher coverage, but only as a (larger) set of single reads.

## Conclusions

With the availability of NGS techniques, studying genetic diversity becomes much quicker and cost-effective as compared to other techniques such as clonal Sanger sequencing or allele specific PCR. Also, additional information not available from these techniques can obtained, provided that the data is analysed properly. ShoRAH reliably reconstructs the local (on the scale of the read length) haplotype structure of a population by correcting sequencing errors with a Bayesian inference algorithm. This approach provides the user also with an estimate of the quality of the reconstruction. Further, ShoRAH can reconstruct the global haplotypes and estimate their frequencies.

ShoRAH has been applied to 454 data from HIV infected patients [4] and Illumina data from tumour dissections (Gerstung *et al.*, in preparation). We foresee its application in the context of viral infections, as well as in bacterial communities and in tumor samples, where the genetic heterogeneity of the sample must be assessed.

## Availability and requirements

ShoRAH (Short Reads Assembly into Haplotypes) is distributed under GNU GPL Licence as C++, Python (< 3.0) and Perl source code and is available from its home page http://www.cbg.ethz.ch/software/shorah. It has to be compiled and run from command line, instructions to install/execute can be found at the online documentation page https://wiki-bsse.ethz.ch/display/ShoRAH/Documentation. It has been tested on Linux and Mac OS X platforms. Other requirements are Biopython, GSL (GNU Scientific Library). The program needle from EMBOSS is required for the alignment only. Working with SAM alignment requires other standard tools not included in the package (SAMtools and pysam) but available from the Web.

## Authors' contributions

OZ developed the error correction model, wrote the local analysis software, published the package and drafted the manuscript. AB developed several optimisation procedures and implemented them. NE and NB developed and implemented the first version of the error correction, the current versions of global haplotype reconstruction and frequency estimation. NB coordinated the project. All authors read and approved the final manuscript.

## Funding

This work has been supported by the Swiss National Science Foundation under Grant No. CR32I2_127017.
